# Haemosporidians from a Neglected Group of Terrestrial Wild Birds in the Peruvian Amazonia

**DOI:** 10.1007/s10393-022-01612-9

**Published:** 2022-08-27

**Authors:** Merit González-Olvera, Arturo Hernandez-Colina, Jocelyn G. Pérez, Gabriela M. Ulloa, Stephanie Montero, Jorge L. Maguiña, Andrés G. Lescano, Meddly L. Santolalla, Matthew Baylis, Pedro Mayor

**Affiliations:** 1 Institute of Infection, Veterinary and Ecological Sciences, University of Liverpool, IC2 Liverpool Science Park, 146 Brownlow Hill, Liverpool, L3 5RF UK; 2 Programa de Pós-Graduação em Saúde e Produção Animal na Amazônia, Universidade Federal Rural da Amazônia (UFRA), Belém, Pará, Brazil; 3 Grupo Enfermedades Emergentes, Universidad Científica del Sur, Lima, Peru; 4 Emerge, Emerging Diseases and Climate Change Research Unit, School of Public Health and Administration, Universidad Peruana Cayetano Heredia, Lima, Peru; 5 Health Protection Research Unit in Emerging and Zoonotic Infections, University of Liverpool, Liverpool, UK; 6 Departamento de Sanitat i Anatomia Animals, Universitat Autònoma de Barcelona, 08193 Bellaterra, Spain; 7 ComFauna, Comunidad de Manejo de Fauna Silvestre en la Amazonía y en Latinoamérica, 332 Malecón Tarapacá, Iquitos, Peru; 8 Coventry, UK

**Keywords:** Amazonia, Haemosporidia, *Haemoproteus*, *Plasmodium*, Terrestrial birds, Subsistence hunting

## Abstract

Haemosporidians are a widespread group of blood parasites transmitted by vectors. Despite their relevance for bird conservation, few studies have been conducted in the Amazonia and even less in terrestrial wild birds. We analysed blood samples from 168 game birds, collected from 2008 to 2015 by subsistence hunters of an indigenous rural community in the Peruvian Amazonia. DNA was tested for *Haemoproteus* spp., *Plasmodium* spp. and *Leucocytozoon* spp. and positive amplicons were sequenced and curated for phylogenetic analysis. Haemosporidian prevalence was 72% overall, 66.7% for *Haemoproteus* spp. and 5.4% for *Plasmodium* spp. and respectively by bird species: Spix’s Guan (*Penelope jacquacu,* *n* = 72) 87.5% and 0%, Razor-billed Curassow (*Mitu tuberosum,* *n* = 45) 77.8% and 6.7%, White-winged Trumpeter (*Psophia leucoptera,* *n* = 20) 6.3% and 12.5%, Blue-throated Piping-guan (*Pipile cumanensis,* *n* = 16) 73.3% and 6.7%, and Great Tinamou (*Tinamus major,* *n* = 15) 10% and 15%. *Leucocytozoon* spp. was not found. *P. leucoptera* and *T. major* were less likely to be infected with *Haemoproteus* spp. Fruit abundance had a negative association with *Haemoproteus* spp. prevalence and precipitation was negatively associated with *Plasmodium* spp. prevalence. The 106 sequences examined represented 29 lineages, 82.8% of them were new lineages (*Plasmodium* *n* = 3, *Haemoproteus* *n* = 21). Novel host-parasite associations and lineages were unveiled, including probably new species of *Plasmodium* spp. Our results highlight the scientific value of alternative sampling methods and the collaboration with local communities.

**Supplementary Information:**

The online version contains supplementary material available at 10.1007/s10393-022-01612-9.

## Introduction and Purpose

The Amazon rainforest is renowned for its high biodiversity, which has not been fully recorded yet, since new species are being described constantly (Metcalf et al. 2020). The Peruvian Amazonia has the second largest portion of the Amazon rainforest (Marzal et al. 2015) and it is one of the most biodiverse regions in the world (Metcalf et al. 2020), its ornithological fauna represents 20% of bird diversity worldwide and, due to their high degree of endemism and risk of extinction, many of these bird species are a conservation priority (Marzal et al. 2015).

Haemosporidian parasites have important effects on their hosts like reducing survival, body condition and reproductive success (García-Longoria et al. 2014; Bush and Clayton 2018). These parasites may constitute another conservation threat for wild birds (Sijbrandra et al. 2017; Chaisi et al. 2019), particularly the lineage SGS-1 of *Plasmodium relictum*, which was recorded for the first time in Peru bordering the Amazonia as an invasive species (Marzal et al. 2015). *Plasmodium* spp., *Haemoproteus* spp. and *Leucocytozoon* spp. are the most common genera of avian haemosporidian parasites on a global scale; however, their prevalence varies according to environmental factors (i.e., altitude, wetland cover, forest cover, vegetation density) and host traits (i.e., body mass, foraging habits, bird species richness, migration distance), across different regions (Fecchio et al. 2021). The latest synthesis on haemosporidian prevalence shows prevalence variations for *Haemoproteus* spp. (7.1–38.4%), *Plasmodium* spp. (5.8–28.6%), and *Leucocytozoon* spp. (0–30.1%) across zoogeographical regions (Fecchio et al. 2021). In the Amazonia, prevalence and diversity of these parasites have marked differences due to climatic conditions (Fecchio et al. 2017, 2018c). For instance, *Plasmodium* spp. and *Haemoproteus* spp. species and lineages were highly diverse and dispersed (Fecchio et al. 2017, 2018a); whereas a single lineage of *Leucocytozoon* spp. has been recorded at low prevalence in one bird species (Fecchio et al. 2018c). Haemosporidian lineages in the Amazonia coexist in the same bird communities presenting different levels of host prevalence and specialization, ranging from generalists to specialists (Svensson-Coelho et al. 2013; Fecchio et al. 2017, 2018a, 2019). Therefore, habitat choice, migratory behaviour and coloniality could affect the prevalence of blood parasites in birds (García-Longoria et al. 2014).

Rainforest game birds are vital links in the complex dynamics of Neotropical rainforest systems and provide key ecosystem services; additionally, they represent a traditional resource for many indigenous cultures for various purposes including a dietary source (Whitworth et al. 2018). Despite their key role, these birds are highly threatened, in particular, large terrestrial birds such as trumpeters (Psophiidae), various cracids (Cracidae) and tinamous (Tinamidae) are the first birds to be hunted in the proximities of human settlements (Lloyd 2004).

In the Peruvian Amazonia, cracids are threatened due to unsustainable hunting practices, and populations of *Mitu tuberosum*, *Penelope jacquacu* and *Pipile cumanensis* have been reduced by over 90% in heavily hunted areas (Begazo and Bodmer 1998). As these birds have intrinsically low rates of reproduction and are dependent on undisturbed forest, their populations cannot be maintained under high hunting pressure (Barrio 2011). According to the Red List of the International Union for Conservation of Nature (IUCN 2021), globally, *Tinamus major* and *Psophia leucoptera* are classified as nearly threatened and *P. jacquacu*, *M. tuberosum* and *P. cumanensis* remain at least concern.

Most of the research on avian haemosporidians has been carried out employing mist nets; hence, it describes the situation exclusively in aerial birds and information about terrestrial birds is minimal. The study of haemosporidian parasites in the Amazonia is beset by logistical and financial restrictions, and information regarding the diversity of their vertebrate hosts and the environmental influence on their prevalence and distribution is incomplete. Therefore, this study aims to characterize haemosporidian infections in terrestrial game birds in the Peruvian Amazonia by exploring parasite diversity, host-parasite associations, prevalence, environmental influences, and parasite phylogeny. In the Amazonia, local communities rely on subsistence hunting for food and could become active samplers of valuable biological material that is usually discarded. To overcome accessibility and conventional fieldwork constraints, this study used blood samples from game birds sustainably hunted over eight years by the indigenous Yagua community of Nueva Esperanza, Peru.

## Methods

### Study Area

**Figure 1:**
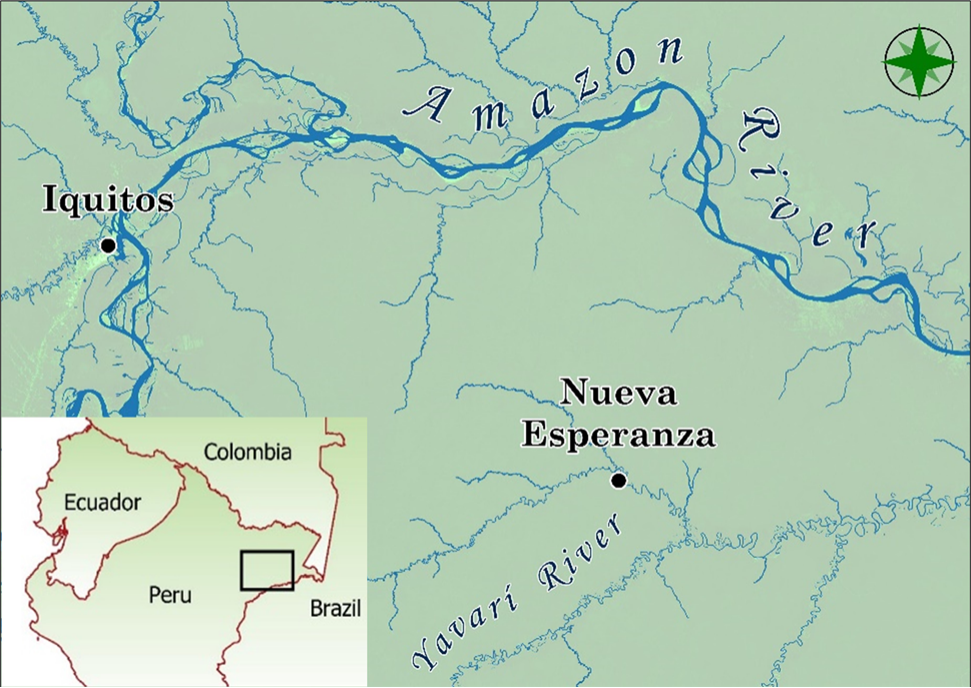
Map of the study area in the territory of the local community of Nueva Esperanza in the Yavarí-Mirín River in the Peruvian Amazonia.

The sampling area is located around the indigenous Yagua community of Nueva Esperanza (04° 19′ 53″ S; 71° 57′ 33″ W; UT5:00), comprised of 281 people (2015) and established in the Yavarí-Mirín River, within the Loreto state in Peru (Fig. 1). The area is composed of 322,500 ha of continuous predominantly non-flooding *terra firme* forest; in 2010, the total disturbed area was 0.8%, mostly caused by logging camps and agriculture (Mayor et al. 2015). Main occupations of local people are small-scale agriculture, fishing, logging, and subsistence hunting within a hunting area of approximately 42,200 ha (Mayor et al. 2015). The climate is typically equatorial with an annual temperature of 22–36°C, a relative humidity of 80–100%, and an annual rainfall of 1500–3000 mm (El Bizri et al. 2018).

### Sample Collection

From 2008 to 2015, blood samples from 168 terrestrial wild birds were collected by subsistence hunters as part of a wildlife conservation program. A total of 168 game birds belonging to three orders (*Galliformes*, *Gruiformes*, *Struthioniformes*), three families (*Cracidae*, *Psophiidae*, *Tinamidae*) and five species were collected by the Yagua hunters. The catches per bird were: Spix’s Guan (*P. jacquacu*) (*n* = 72), Razor-billed Curassow (*M. tuberosum*) (*n* = 45), Great Tinamou (*T. major*) (*n* = 20), White-winged Trumpeter (*P. leucoptera*) (*n* = 16), and Blue-throated Piping-guan (*P. cumanensis*) (*n* = 15) (Table S1). Local hunters were asked to impregnate blood from the cranial or caudal cava vein of the hunted birds on either Whatman filter paper No. 3, FTA® cards or protein saver cards. Samples were collected in all seasons, and hunters recorded the species, date, location, and sex in the case of sexual dimorphism. Cards were placed individually in tagged paper envelopes and kept at environmental temperature protected from light and humidity until sent for storage (− 20°C) and processing at the EmergeLab, LID 412 of Universidad Peruana Cayetano Heredia.

### Molecular Methods

Whatman filter paper No. 3, FTA® cards or protein saver cards were placed into a petri dish and a 6 mm^2^ piece was cut into smaller pieces with a scalpel. Prior to DNA extraction, sample preservation condition was recorded (good, medium, bad) as well as fungal presence (abundant, present, scarce, none). The extraction was done using a QIAamp® DNA Mini Kit following the manufacturer´s instructions, except for the incubation, which elapsed overnight to increase the DNA yield. Extracted DNA was tested for the most common haemosporidians (*Haemoproteus* spp., *Plasmodium* spp. and *Leucocytozoon* spp.) following the protocol by Hellgren et al., (2004), which consists of a nested PCR that amplifies a 479-bp fragment of the cytochrome b (*cytb*) gene. In the first part of the reaction 1 µl of DNA template, 1 µl of forward primer HaemNF1, 1 µl of reverse primer HaemNR3, 10 µl of Bioline mix, 1 µl of BSA (bovine serum albumin), and 6 µl of nuclease-free water were mixed to reach a final volume of 20 µl. The PCR profile was 22 cycles at 94°C for 3 min, 94°C for 30 s, 50°C for 30 s, 72°C for 45 s, followed by a final extension at 72°C for 10 min. For the second part, 2 µl of PCR product from the previous reaction were used as template, 10 µl of MyTaq™ Red MixPCR master mix, 1 µl of BSA and 5 µl of nuclease-free water were employed. The samples had a DNA concentration range of 50–700 ng/µl (median 200 ng/µl), all primers were used at a 10 µM concentration, and BSA was used at a 0.5% concentration. For detection of *Plasmodium* spp. and *Haemoproteus* spp., 1 µl of forward primer HaemF and 1 µl of reverse primer HaemR2 were added, whereas for the detection of *Leucocytozoon* spp. 1 µl of forward primer HaemFL and 1 µl of reverse primer HaemR2L were used. The PCR profile for the second part was 36 cycles at 94°C for 3 min, 94°C for 30 s, 50°C for 30 s, 72°C for 45 s, followed by extension at 72°C for 10 min. Molecular grade water was used as a negative control and genomic DNA from *P. bergei* ANKA or genomic DNA from *Leucocytozoon* spp. was used as a positive control. The amplicons were visualized on a 1.5% agarose gel with SYBR™ Safe DNA gel satin (Thermo Fisher Scientific). Positive PCR products were sent for sequencing in forward and reverse direction with the Sanger method employing the primers HAEMF and HAEMR2. Resulting sequences were visualized, assembled, and manually corrected using BioEdit. Sequence reads were compared to previously published avian Haemosporidia in the GenBank nucleotide database using BLASTn to identify the genus of the parasite. Mixed sequences (*n* = 6), indicated by electropherograms with consistent double peaks in the same base position, were considered as mixed infections (Lutz et al. 2015) and were excluded from further analysis.

### Sequence Clustering

Sequence clustering and phylogenetic analysis were used to relate each observed sequence to all previously published sequences. To guarantee the most thorough comparison of new sequences with those recorded previously, a randomly chosen *Haemoproteus cytb* sequence, was used for the GenBank search. The query showed 10,000 matches ranked in descending sequence identity, which included non-avian *Plasmodium*, *Haemoproteus,* and *Leucocytozoon* sequences at the lower end, ensuring that all available avian *Plasmodium* and *Haemoproteus* sequences were comprised. All avian *Plasmodium* and *Haemoproteus* sequences from the query were selected and joined with an additional 224 voucher sequences from MalAvi. After removing duplicates, partial sequences, and sequences containing ambiguous bases, the reference sequences were aligned in Bioedit (Hall 1999) using ClustalW (Chenna et al. 2003) with all new sequences identified in this study (*n* = 106) to produce a 475 bp multiple sequence alignment (*n* = 5810). To simplify the phylogeny, CD-HIT was used to cluster sequences into common lineages, defined by a 1% sequence divergence threshold, as this value retained the best morphospecies of the reference lineages used. Sequences found in the same cluster were considered to be the same lineage. Hence, Peruvian sequences found in clusters with previously described lineages were identified as such, and sequences in clusters without reference lineages were established as new lineages and were named following the standard protocol of Bensch et al. (2009), creating a six-letter code formed by the first three letters of both, the host genus and species, followed by a number to denote multiple lineages from a single host species. New lineages were deposited in GenBank under the accession numbers MZ614915–MZ614938 and ON246344.

### Phylogenetic Analysis

To estimate a phylogeny of our sequences, representative reference sequences from the clusters identified in the CD-HIT analysis, and representative sequences of each new lineage from our collection were employed. The sequences were aligned using ClustalW [33] and *Leucocytozoon* was used as outgroup to root the tree. A Maximum Likelihood (ML) phylogeny was estimated using MEGA X (Kumar, et al. 2018) with a GTR + G + I nucleotide substitution model (estimated by Smart Model Selection using the Akaike Information Criterion). The data set was partitioned to allow independent modelling of base substitution rates at each codon position, a measure that will reduce the effect of homoplasy at the third position. Node robustness was evaluated with an SH-like log-Likelihood ratio test. The software FigTree v1.3.1 was used to visualize the phylogenetic tree. Sequences in clades containing sequences derived from a named morphospecies were assigned to that morphospecies.

### Data Analysis

We analysed (1) the biological susceptibility, including bird species, family, and sex, and (2) the environmental drivers of infection, with the year, precipitation, season, temperature, river level and ripe fruit availability as explanatory variables. Precipitation and river level values were obtained from the HidroWeb portal of the National System of Water Resources Information (SNIRH) of Brazil. The data from the four closest stations to the study area in the Yavari River with complete records of the day the birds were hunted were used. We classified the months according to their average precipitation over the years of our sampling. The months that received more rain than the overall average + 1 standard deviation (SD) were classified as rainy months, the ones that received less than the average—1 SD as dry months and those between ± 1 SD as intermediate months (normality was confirmed with a Shapiro–Wilk normality test, *P* = 0.452). In this way, the rainy months were January and March (294–329 mm), the intermediate months were February, May–June, October–December (165–294 mm) and the dry months were July–September (142–165 mm). The temperature data was obtained from the NCEI Climate Data Online service requesting the daily summaries for Iquitos station (Station ID: GHCND:PE000084377) given that it is the closest to the sampling area (approximately 195 km). As the minimum and maximum temperature records were incomplete, only the daily average temperature was extracted and then averaged by month for analysis. In all cases, the search period was from the 1st of January 2008 to the 31st of December 2015.

Body condition and fitness of individuals have been shown to influence their immune response against parasitic, viral, and bacterial infections (van Hoesel et al. 2020). Thus, we used seasonal fruit availability to relate the environmental stress with infection rates in these frugivorous bird species. The percentage of ripe fruit in the study area was obtained from El Bizri et al. (2018), were the authors observed the canopy of trees and vines monthly along two transects in upland forest and one in upland swamp forest dominated by palms; the length and area of each transect were determined by the discovery rate of new species and as it plateaued, it was considered representative of fruit abundance in the area.

Generalized linear models (GLM) were used to analyse the prevalence and number of lineages of *Haemoproteus* and *Plasmodium*, separately. We used logistic regression to estimate parasite prevalence. All combinations of variables were explored, but variables that are not independent of each other or that showed collinearity were not included in the models simultaneously (non-independent variables: family and species; colinear variables: river level, precipitation, season, and fruit abundance in upland forest and in swamp forest; variance inflation factors > 3). As the number of infected birds was correlated with the number of lineages found by bird species (Pearson’s correlation, *r*^2^ = 0.999, *P* < 0.001), the number of lineages in relation to the biological susceptibility was not analysed as the results were very similar to the ones for the parasite prevalence. However, the number of lineages of *Haemoproteus* spp. in relation to environmental variables was aggregated by month and analysed using a negative binomial regression model. The number of *Plasmodium* spp. lineages was insufficient for analysis.

The influence of the card types for blood storage (Filter paper W3, FTA® cards and Protein saver cards), sample preservation (good, medium, bad) and fungal presence (abundant, present, scarce, none) on the test result was assessed using logistic regression; all variable combinations were explored.

For all models, observations without complete data for all variables were excluded. Candidate models were selected using the Akaike’s Information Criterion (AIC) theoretic approach and the ones within two AIC units (i.e., ΔAIC < 2) were considered as the best-supported by the evidence in the data. The second order AIC (AICc) was used instead for the models assessing the number of lineages to account for the small sample size (*n* = 37). The variables used in each model, AIC, ΔAIC and AIC weights are shown in Supplementary Tables S3–S6. Given *P* values are for the best-supported models. All analyses were performed in R (version 4.1.1).

### Ethics

All applicable institutional and national guidelines for the care and use of animals were followed. Birds were killed exclusively as part of the usual activities of local hunters. The research protocol and export permits were approved by Servicio Nacional Forestal y de Fauna Silvestre of Peru (Peruvian Forestry and Wildlife Agency) (Research Ethics Committee for Experimentation in Wildlife Protocols resolutions 041-2007- DGGFS-DGEFFS, 0350-2012-DGFFS-DGEFFS, 258-2019-MINAGRI-SERFOR-DGGSPFFS, and export permit 003457-SERFOR) and the Institutional Animal Use Ethics Committee from Universidad Peruana Cayetano Heredia (resolution 029-03-19, protocol 102142).

## Results

**Figure 2:**
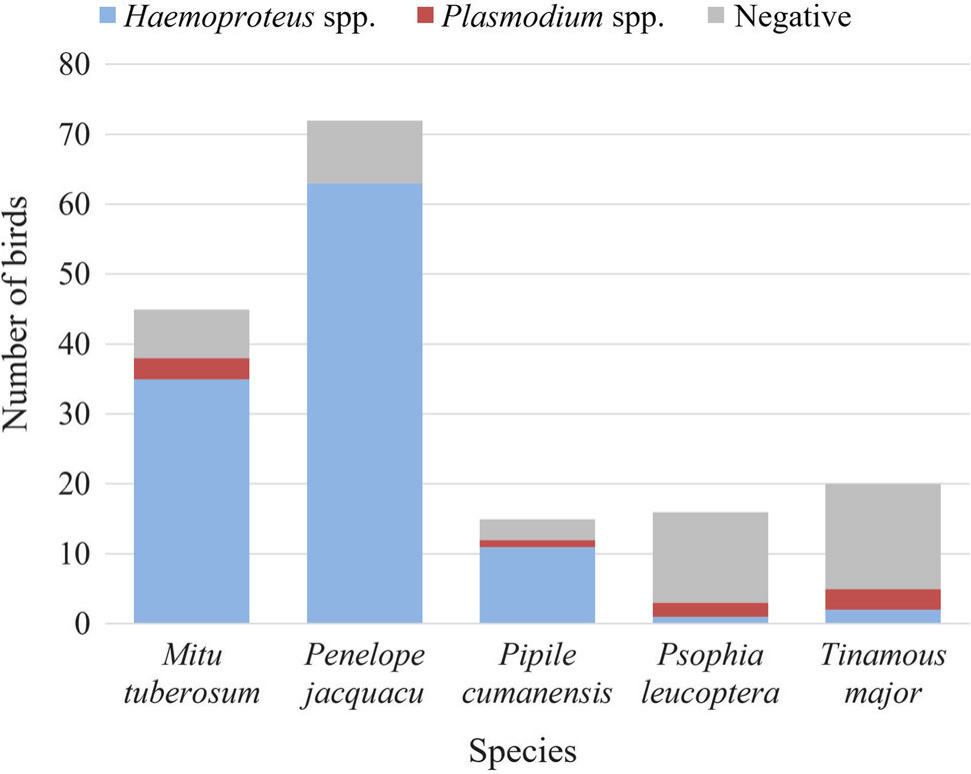
Distribution of terrestrial bird species studied (*n* = 168) according to infection by *Haemoproteus* spp. and *Plasmodium* spp. in the territory of the local community of Nueva Esperanza in the Yavarí-Mirín River. *P. leucoptera* and *T. major* were less likely to be infected by *Haemproteus* spp. (*P* < 0.001).

The haemosporidian prevalence was 72% overall, for *Haemoproteus* spp. it was 66.7% (112/168) and for *Plasmodium* spp. it was 5.4% (9/168). *Leucocytozoon* spp. was not found. *Haemoproteus* spp. prevalence was higher in *P. jacquacu* (87.5%, 63/72), *M. tuberosum* (77.8%, 35/45) and *P. cumanensis* (73.3%, 11/15); whereas the highest *Plasmodium* spp. prevalence was found in *T. major* (15%, 3/20), and *P. leucoptera* (12.5%, 2/16) (Fig. 2). For the assessment of the biological susceptibility, the highest supported models included family or species; nevertheless, the model with the lowest AIC was the one for species alone, which showed a significant negative association between *Haemoproteus* spp. prevalence and *P. leucoptera* (*P* < 0.001) and *T. major* (*P* < 0.001). No significant effects were observed in the supported models for *Plasmodium* spp. Regarding sample storage, the best models showed that FTA® cards had higher positivity (*P* = 0.002), whereas sample preservation and fungal contamination were not associated with positivity differences.

**Figure 3:**
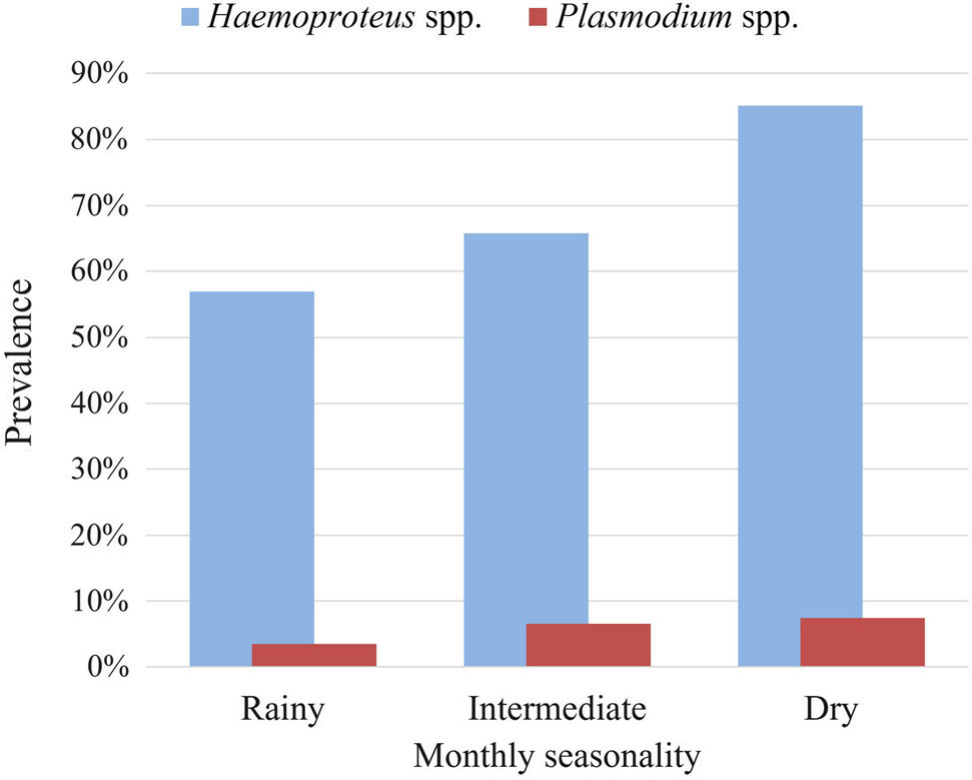
Overall Haemosporidian prevalence in terrestrial birds (*n* = 168) in the territory of the local community of Nueva Esperanza in the Yavarí-Mirín River by monthly rain seasonality. Rainy: January and March (294–329 mm); Intermediate: February, May–June, October–December (165–294 mm); Dry: July–September (142–165 mm).

**Figure 4:**
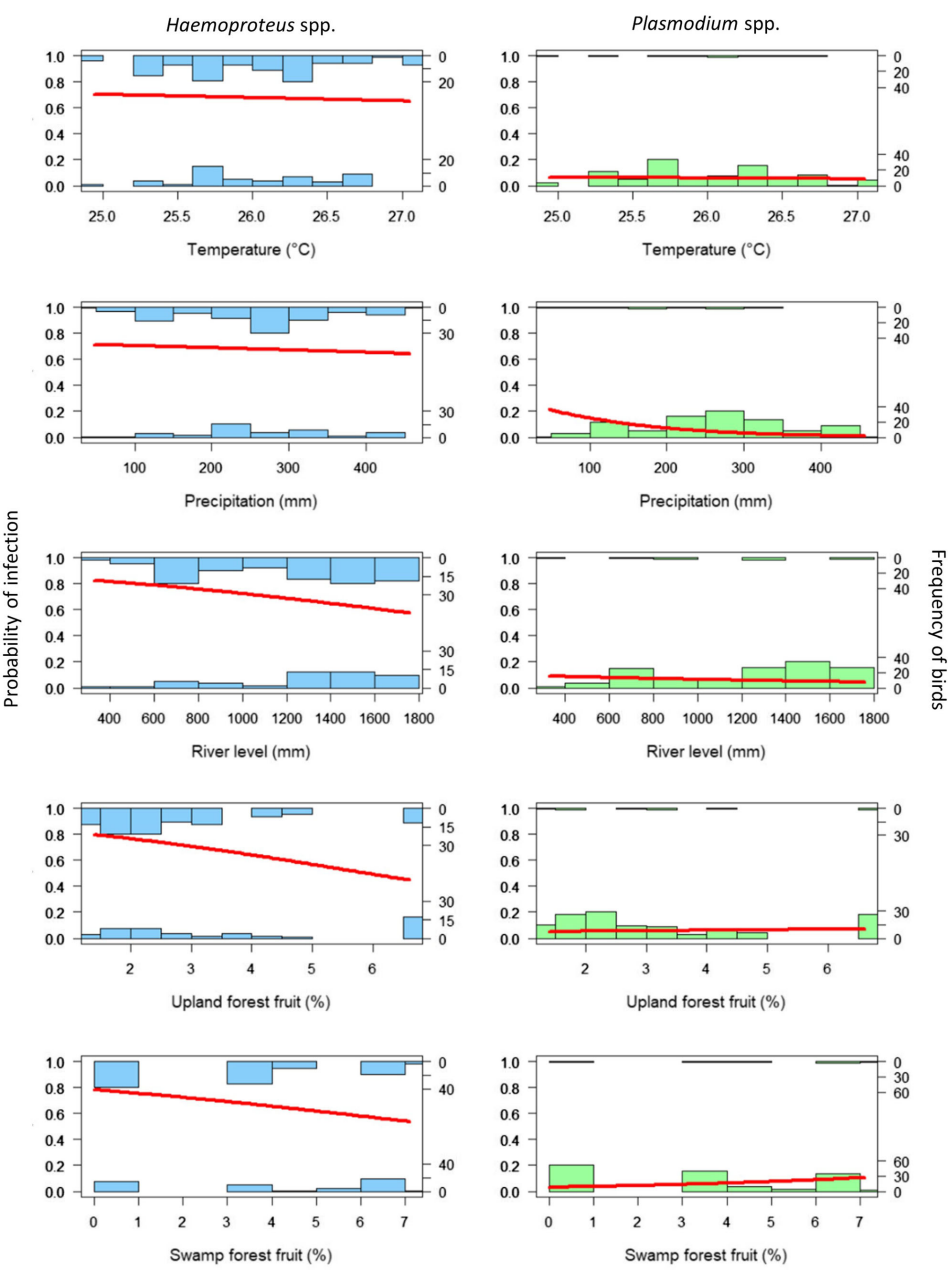
Association between environmental variables and Haemosporidian prevalence in terrestrial birds (*n* = 168) in the territory of the local community of Nueva Esperanza in the Yavarí-Mirín River. Regression curves were constructed for each variable independently. Upper histogram shows positive individuals and lower histogram shows negative individuals. For *Haemoproteus* spp., the percentages of fruit in upland forest was significant (*P* = 0.002), and for *Plasmodium* spp. precipitation was significant (*P* = 0.032).

*Haemoproteus* spp. infections were observed in all years with the highest prevalence in 2013 (88.5%, 23/26) and the lowest in 2008 (40.9%, 9/22). *Haemoproteus* spp. prevalence was significantly higher in 2013 (*P* = 0.017) and 2015 (*P* = 0.04). *Plasmodium* spp. infections were not observed between 2008 and 2014; the highest prevalence was observed in 2011 (12.5%, 1/8) and the lowest in 2009 (3.7%, 1/27) (Table S1). For the environmental influence on *Haemoproteus* spp. infection, the three best-supported models indicated a negative association with the abundance of fruits in upland forest habitats (*P* = 0.002). In the case of *Plasmodium* spp. infection, only the precipitation had a negative relation in the selected models (*P* = 0.032). No significant environmental association was found for the number of *Haemoproteus* spp. lineages and only one model including precipitation was supported. Figures 3 and 4 show parasite prevalence by monthly seasonality and environmental variables, respectively.

### Haemosporidian Genetic Analysis

From the 121 *cytb* sequences obtained in this study, 14 were either mixed, partial, or poor-quality sequences; hence, they could not be further analysed. The remaining sequences were clustered at 1% divergence thresholds since it was observed that it adequately retained morphospecies. A total of 106 *cytb* sequences were identified in this study, 100 within the genus *Haemoproteus* and six within the genus *Plasmodium*. In total, 29 different lineages were identified, 24 (82.8%) were recorded for the first time, three belonged to *Plasmodium* (10.4%) and 21 to *Haemoproteus* (72.4%). The majority of novel lineages were isolated from *M. tuberosum*. Most lineages for both *Haemoproteus* and *Plasmodium* were observed once. The most prevalent lineage (TOFLA03) was recorded on 49 birds from four species with most of its observations coming from *P. jacquacu* (Table 1).

**Table 1:** *Plasmodium* and *Haemoproteus* lineages by bird species from the Nueva Esperanza settlement of the Peruvian Amazonia.

Lineages	Spix’s guan (*Penelope jacquacu*)	Razor-billed Curassow (*Mitu tuberosum*)	White-winged Trumpeter (*Psophia leucoptera*)	Blue-throated Piping-guan (*Pipile cumanensis*)	Great Tinamou (*Tinamus major*)	Total
DENVID01^P^				1		1
*MITTUB02^P^		1				1
*MITTUB03		1				1
*MITTUB04		1				1
*MITTUB05		1				1
*MITTUB06		1				1
*MITTUB07		1				1
*MITTUB08		1				1
*MITTUB09		1				1
*MITTUB10		1				1
*MITTUB11		2				2
*MITTUB12		4				4
*MITTUB13		5				5
*MITTUB14		8				8
*MITTUB15		1				1
PENJAC01	6					6
*PENJAC02	1					1
*PENJAC03	1					1
*PENJAC04	1					1
*PENJAC05	3					3
PENOBS01^b^	6					6
*PIPCUM02				1		1
*PIPCUM03				1		1
*PIPCUM04				1		1
*PSOLEU02^P^			1			1
*PSOLEU03			1			1
PSOOCH01^P^			1			1
*TINMAJ01^P^					3	2
TOFLA03^a^	38	4		6	1	49
Sequences analysed	56	33	3	10	4	106
Found lineages•	7	15	3	5	2	29♦

^a^*Haemoproteus paraortalidum.*

^b^*Haemoproteus ortalidum.*

^P^*Plasmodium* spp. lineages. Other lineages without superscripts belong to *Haemoproteus* spp.

*New lineages found in this study.

•Number of different lineages observed in each bird species.

♦Number of different lineages observed in the birds.

For *Plasmodium,* five different lineages were observed, and three were reported for the first time (Table 1). Previously reported parasite lineages that matched sequences from this study were: TOFLA03 (Accession number JX029916) (*n* = 50), PENOBS01 (Accession number KX171627) (*n* = 6), PENJAC01 (Accession number KF482345) (*n* = 6), and PSOOCH01 (Accession number KU562606) (*n* = 1) and DENVID01 (Accession number KU057966). Three lineages belong to unidentified morphospecies, PENJAC01 (*Haemoproteus*) and PSOOCH01 and DENVID01 (*Plasmodium*), whereas PENOBS01 corresponds to the morphospecies *Haemoproteus ortalidum* and TOFLA03 to *Haemoproteus paraortalidum*. No *Plasmodium* morphospecies could be identified in this study. For the new lineages, the closest related sequences had an identity of 95–99% and a nucleotide correspondence of 444–467 bp out of 469 bp (Table S2).

**Figure 5:**
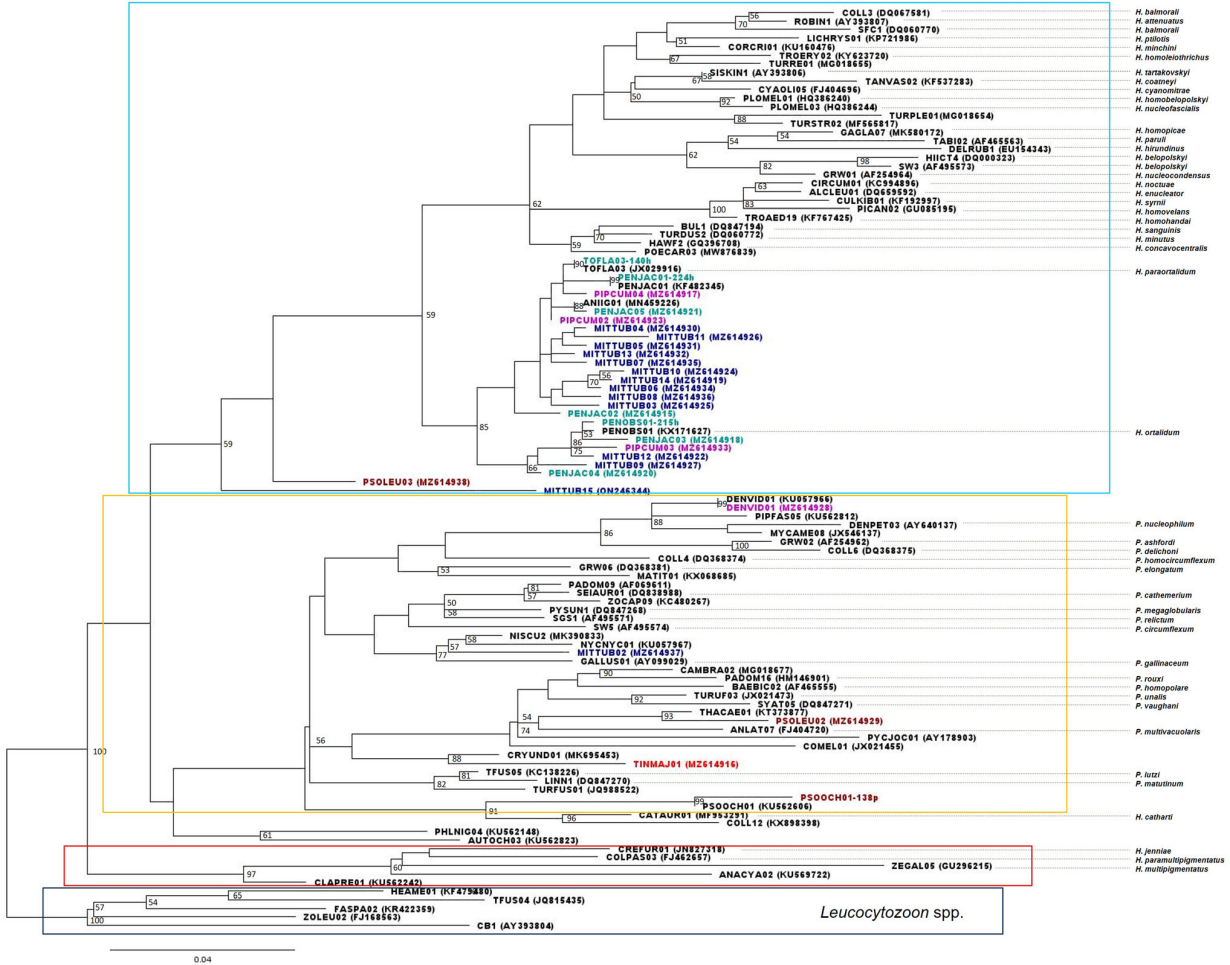
Maximum likelihood phylogeny of the *cytb* sequences obtained in this study and reference sequences. The phylogeny was estimated from a 49 bp multiple sequence alignment using a GTR + $\Gamma$ + I model (α = 0.488; proportion of invariant sites = 0.248). The tree is rooted with an outgroup of *Leucocytozoon* sequences (boxed in black). Node accuracy is indicated by an SH-like log-Likelihood ratio metric; lower bootstrap values (< 50) are omitted. All sequences with different lineage names were obtained with a 1% sequence divergence threshold. Reference sequences obtained from MalAvi and GenBank are shaded in black, the rest of the sequences were obtained from this study and are shaded according to the bird they were obtained from *Penelope jacquacu* (Green), *Pipile cumanensis* (Pink), *Mitu tuberosum* (Blue), *Psophia leucoptera* (Brown) *Tinamous major* (Red). Sequences are named by their lineage, and their accession numbers are indicated in parenthesis. Known parasite species for the lineages are listed to the right. Different parasite genus and subgenus are indicated by boxes: *Haemoproteus* (Parahaemoproteus) (Blue), *Plasmodium* (Yellow), *Haemoproteus* (Haemoproteus) (Red). Branches lengths are drawn proportionally to evolutionary distance. Lineages AUTOCH3, PHLNIG04 and COLL12 are listed as *Haemoproteus* spp. (MalAvi 2021) (Color figure online).

*Plasmodium* sequences formed a single clade in the ML tree and newly described lineages, TINMAJ01, MITTUB02 and PSOLEU02 identified as *Plasmodium* by NCBI blast, were placed in that clade. In addition, the lineages PSOOCH01 and DENVID01, which matched a sequence isolate in this study, were also positioned in the *Plasmodium* clade (Fig. 5). Another two well-supported clades for both *Haemoproteus* subgenus were produced. The Parahaemoproteus subgenus clade formed two distinctive subclades with one of them containing all the new *Haemoproteus* lineages found in this study as well as reference sequences described in the Amazonia. Interestingly, in the *Plasmodium* clade, there were two subclades that contained *Haemoproteus* sequences, one containing only *Haemoproteus* lineages (AUTOCH03 and PHLNIG04) and the other one grouped *Haemoproteus* (CATAUR01 and COLL12) and *Plasmodium* (PSOOCH01) lineages. The lineages PSOLEU03 and MITTUB15, identified as *Haemoproteus* spp., formed a single clade isolated from the other two main *Haemoproteus* clades (Fig. 5).

## Discussion

To our knowledge, this is the first study of haemosporidian infection focused exclusively on wild populations of terrestrial birds. We present the first record for *Haemoproteus* spp. infection in *P. leucoptera* and in *M. tuberosum* and the first report of haemosporidian parasites infecting *T. major* (*Plasmodium* spp.) and *P. cumanensis* (*Haemoproteus* spp. and *Plasmodium* spp.). The only host-parasite association reported before is *Haemoproteus* spp. infecting *P. jacquacu* (MalAvi 2021).

The overall haemosporidian prevalence was 72% and *Plasmodium* spp. prevalence (5.4%) was contrasting with the one for *Haemoproteus* spp. (66.7%). These parasites have been recorded previously in the Amazonia with a combined prevalence between 17.4% (Fecchio et al. 2018c) and 21.7% (Svensson-Coelho et al. 2013), and a similar individual prevalence for *Plasmodium* spp. and *Haemoproteus* spp. of 2.4, 15.3% (Fecchio et al. 2018c) and 6.4, 9.6% (Svensson-Coelho et al. 2013), respectively. Prevalence differences may be due to several effects such as the sampling area, host fitness and bird species. The ecosystem in which this and previous studies were carried out is categorised as *terra firme* forest; however, this is a broad definition of a complex ecosystem (Asner et al. 2013). The Amazonia consists of a mosaic of different habitats and a habitat classification system that considers all minor habitat types occurring in the region is missing (Harder 2000). Habitat type has been described to be associated with parasite host-specialization (Sehgal 2015), and it is known to affect vector populations and host-parasite-vector interactions. In fact, Fecchio et al. (2018c) found significant differences in *Haemoproteus* spp. and *Plasmodium* spp. prevalence amongst six areas of endemism.

Previous studies were based on bird netting, whereas in this study hunted birds were employed. It has been argued that nets catch mainly healthy birds since this method relies on the active movement of birds, and haemosporidian infected birds have shown a decrease in their locomotion (Valkiūnas 2004). Furthermore, Valkiūnas (1991, 1993) demonstrated that birds on the parasitaemia peak are under-sampled by mist netting but can be obtained by other collection methods such as shooting. It was not possible to test if haemosporidian infection was related to hunting probability; however, future studies should integrate different methods to compare haemosporidian prevalence.

Prevalence varies greatly among avian species because of differences in immune response (Calero-Riestra 2016), physiology (Ortego et al. 2008), breeding season (Lachish et al. 2011), and nesting foraging and flocking behaviour (Gonzalez et al. 2014). Haemosporidian prevalence has been evaluated in different avian groups previously (e.g., Krone et al. 2008; Quillfeldt et al. 2011; Gao et al. 2021; Hernandez-Colina 2021), looking for changes associated with their species, life traits and habitats. Terrestrial lifestyle might change the exposure rate to vectors and may also put birds in contact with different vector species compared to aerial birds. To our knowledge, there is only one study reporting haemosporidians in Amazonian terrestrial wild birds, reporting *Plasmodium* spp. infection in *P. leucoptera* (66.7%, 2/3) (Fecchio et. al. 2018b). Chagas et al. (2016) reports one out of six individuals of *Penelope superciliaris* infected with *Plasmodium* spp., this terrestrial bird is present in the Amazonia, but in this case, it was sampled in the Atlantic Forest (Chagas et al. 2016). Other studies reported blood parasites on captive terrestrial birds, Cracidae: *Aburria jacutinga* (*Haemoproteus* spp.; 62.1%, 18/29) (Ferreira-Junior et al. 2018), *A. jacitinga* (haemosporidians; 42.8%, 18/42) (Motta et al. 2013), *A. mantelli* (*Plasmodium* spp.; 78.0%, 25/32) (Banda et al. 2013), *Mitu tormentosum* (*Plasmodium* spp.; 20.0%, 1/5), *Nothocrax urumutum* (*Plasmodium* spp.; 20.0%, 1/5), *Penelope obscura* (*Haemoproteus* spp.; 50.0%, 1/2) and *Pipile jacutinga* (*Plasmodium* spp., 66.7%, 2/3), Phasianidae: *Pavo cristatus* (*Plasmodium* spp.; 12.9%, 4/31), *Pavo muticus* (*Plasmodium* spp.; 25.0%, 1/4), and Sthrutionidae: *Struthio camelus* (*Plasmodium* spp.; 12.5%, 1/8) (Chagas et al. 2017).

Important differences were observed in parasite prevalence by bird species, which has also been reported in Western Amazonia (Svensson-Coelho et al. 2013). Here, *P. leucoptera* and *T. major* were unlikely to be infected by *Haemoproteus* spp. compared to the other species, but no significant influences were found for *Plasmodium* spp. infection possibly due to the low number of infected birds. Life traits may influence parasite prevalence, like in the Ecuadorian Amazonia, where differences in prevalence by foraging height were found (Svensson-Coelho et al. 2013). Although all the birds studied here share terrestrial habits, their nesting, grouping, and feeding behaviours are different. For instance, *P. jacquacu* shows partial arboreous behaviour (Mogollón 2016), whereas *T. major* remains mostly on ground level (Guerta and Cintra 2014), and *P*. *cumanensis* are capable of gliding for long distances (Mogollón 2016) giving them the ability to widen their geographic range.

Bird species with high *Haemoproteus* spp. prevalence had low *Plasmodium* spp. prevalence and vice versa. That same pattern has already been recorded in Amazonian birds (Svensson-Coelho et al. 2013) and it could be indicative of vector host preferences, particularly since each genus is vectored by different dipteran insects (Ferreira-Junior et al. 2018). Therefore, exposure rates could be also associated with bird behaviours (nesting type and height, social behaviour, foraging height, etc.) (Svensson-Coelho et al. 2013). Indeed, Fecchio et al. (2018b) found that infection and diversity of *Plasmodium* responded mainly to geography, while *Haemoproteus* was related to host associations. *P. jacquacu* was the most represented bird in our sampling (*n* = 72), it had the highest haemosporidian prevalence (87.5%) and was infected exclusively with *Haemoproteus* spp. Hence, either it is refractory to *Plasmodium* spp. due to its biology or behaviour, or it is infected at such low prevalence that further sampling is required to uncover this association.

Climatic variables influence the life cycle of haemosporidian and their vectors, and in consequence could affect parasite transmission (Santiago-Alarcon et al. 2012). However, no significant positive effects were observed in relation to variables typically related to prevalence (i.e., temperature and precipitation) possibly because in the Amazonia these variables are maintained within the range that favours vectors’ life cycle and, therefore, parasite transmission (Fecchio et al. 2017). Precipitation can be a predictor of mosquito abundance and distribution and therefore, related to the prevalence of avian malaria (Sehgal 2015). Nonetheless, the negative association of precipitation with *Plasmodium* spp. prevalence observed could be due to the limiting effect of rain on mosquito flying activity and consequently host-seeking behaviour; besides, heavy rain can reduce mosquito survival (Karki et al. 2016).

We found a negative association between fruit abundance and *Haemoproteus* spp. prevalence, and to our knowledge, the influence of food resources and Haemosporidian prevalence has not been explored before. The diet of the bird species sampled here is mainly based on fruits and complemented with invertebrates, seeds, leaves and flowers (Mogollón 2016; Schelsky 2004; Sherman 1995). The availability of resources is related to body condition and fitness of individuals and consequently, their immune response (van Hoesel et al. 2020). Therefore, when fruits are scarce, birds’ immune response could be suboptimal, increasing susceptibility to *Haemoproteus* infection.

In the phylogeny, matches to reference sequences from our *Haemoproteus* samples were exclusive with sequences reported in the Amazonia, suggesting that geographical barriers have impeded the introduction of exotic parasites to this geographic area (Chagas et al. 2017). *Haemoproteus* sequences clustered with sequences of the Parahaemoproteus subgenus and originated from a main branch suggesting that they are highly related. This could be due to their speciation history since these parasites showed low rates of speciation events, and parasites from the Parahaemoproteus subgenus are known to have high host specificity (Fecchio et al. 2018a).

Contrary to the *Haemoproteus* clustering in the phylogeny, our *Plasmodium* sequences grouped apart from each other, and we observed only two matches, one with a lineage previously recorded in the Amazonia (PSOOCH01) and one with a lineage obtained in the Brazilian Atlantic Forest (DENVID01). We included other sequences isolated from the Amazonia in related birds to the ones we studied (MYCAME08 and NYCNYC01); however, they did not cluster together or with our sequences. Noteworthy is the clade formed by PSOOCH01, CATAUR01 and COLL12, as it is composed of one *Plasmodium* sequence and two *Haemoproteus* sequences and it is placed in the *Plasmodium* clade. Nevertheless, in this phylogeny, the *Plasmodium* clade has the lowest nodal support; moreover, it has been suggested that PSOOCH01 and CATAUR01 may correspond to a new genus of haemosporidian (Vanstreels et al. 2022), which may explain this odd placing. Haemosporidian phylogenies constantly present inconsistencies given the high diversity of these parasites and the lack of studies in certain bird species and areas; nonetheless, addition of new sequences contributes to the shaping of phylogenies and to shed light on the evolutionary origin of these parasites.

It is possible that some of the new lineages observed in this study, either for *Haemoproteus* or *Plasmodium*, represent new species, particularly those with the higher nucleotide divergence compared to reported lineages and those whose tree placing is in isolated branches with high nodal support. However, the lack of morphological evidence and the unresolved relationships precludes us from confirming if new species have been found.

Some of the sequences obtained in this study clustered with lineages from GenBank and MalAvi (TOFLA03, PENOBS01, PENJAC01 andPSOOCH01). The lineage TOFLA03, belonging to *H. paraortalidum*, was the most frequently observed in this study. This lineage was previously observed in Peru and Brazil on Galliformes and Passeriformes (MalAvi 2021). The lineage PENOBS01 of the *H. ortalidum* species has been previously reported in Brazil in birds of the Galliformes order (Chagas et al. 2017). The lineage PENJAC01 was reported in Peru (MalAvi 2021), DENVID01 was isolated from the Brazilian Atlantic Forest (Chagas et al. 2016), and PSOOCH01 (*Plasmodium* spp.) was recorded in the Brazilian Amazonia (Fecchio et al. 2017). Despite evidence of migrant birds in the area (Harvey et al. 2014) and the recent discover of invasive haemosporidian species in Peru (Marzal et al. 2015), we found no proof of foreign parasites since the reference lineages have only been reported in the mentioned bird orders and locations. The rest of the sequences obtained here represented new lineages. Three new lineages were recorded for *Plasmodium* and 21 for *Haemoproteus*, corresponding to 82.8%; a similar proportion of new lineages has also been reported for the Brazilian Amazonia (91.4%) (Fecchio et al. 2018b). Neotropical regions, such as the Amazonia, are considered as hotspots of avian diversity and the positive association between host and haemosporidian diversity indicates that the diversity of the latter could be also great and that new species and lineages are likely to be discovered as more studies are performed in the area (Chagas et al. 2017). Most of the new lineages were found in a single bird species (*M. tuberosum*) suggesting that they are specialists. Nevertheless, it should be considered that most of the lineages retrieved here had only one observation and they might be found in more bird species over time. Conversely, the lineage TOFLA03 may be a generalist since it was found on four of the five bird species and for eight years. Most of the new lineages were isolated from *M. tuberosum* (MITTUB03–MITTUB15), this might suggest that this bird is exposed to a more diverse array of vectors due to its biological or behavioural characteristics. For instance, *M. tuberosum* is reported to be the bird with the largest distribution range amongst the ones collected here (Mogollón 2016), and birds with larger ranges may experience a greater risk of infection by more parasite species (García-Longoria et al. 2014; Fecchio et al. 2017).

Of the three different sampling cards used in this study, FTA® cards produced a better testing success rate, thus their use is recommended for future studies. Although we did not observe interference by fungal contamination or sample preservation, samples should be preserved as best as possible.

Future research should include clinical and histopathological examinations to define health implication of haemosporidian and blood smears to complement the classification of these parasites by morphology. Also, studying dipteran vectors present in the area, their competence, abundance, and host preferences would allow to assess the environmental influence on their life cycles. Together, this information will help us to understand the role of these parasites as an additional pressure on the conservation of threatened species.

## Conclusion

This is one of the first studies to demonstrate high haemosporidian infection rates in populations of terrestrial birds from remote areas in the Amazonia. The participation of the local community has been key to providing a large set of blood samples from species that are usually not studied. We also showed that climatic variables and fruit availability influence the probability of infection. Various lineages and host-parasite associations were recorded for the first time showing that there is a great diversity in Amazonian haemosporidian and that more studies are required to fully describe it. Reporting the distribution and diversity of avian blood parasites in the Peruvian Amazonia is an important first step to understand the natural parasite distribution and prevalence in relation to environmental variables.

## Supplementary Information

Below is the link to the electronic supplementary material.

Supplementary file1 (DOCX 38 KB)

## Data Availability

All data generated during this study are included in this published article and its supplementary information file. Environmental data are available from the corresponding author on reasonable request.
