# Supplementary material for: Haemosporidians from a Neglected Group of Terrestrial Wild Birds in the Peruvian Amazonia
Source: Ecohealth. 2022 Aug 27;19(3):402–16. doi: 10.1007/s10393-022-01612-9 (PMC9573858; doi:10.1007/s10393-022-01612-9)
Supplement: Supplementary file 1 — Supplementary file1 (DOCX 38 KB) [file 10393_2022_1612_MOESM1_ESM.docx]

## **Supplementary Tables**

**Table S1.** Number of birds collected (n = 168) by species and year in the territory of the local community of Nueva Esperanza in the Yavarí-Mirín River in the Peruvian Amazon.

| **Species** |  | **2008** | | | **2009** | | | **2010** | | | **2011** | | | **2012** | | | **2013** | | | **2014** | | | **2015** | | | **NR** | | | **Total** | | |
| --- | --- | --- | --- | --- | --- | --- | --- | --- | --- | --- | --- | --- | --- | --- | --- | --- | --- | --- | --- | --- | --- | --- | --- | --- | --- | --- | --- | --- | --- | --- | --- |
|  |  | **H** | **P** | **T** | **H** | **P** | **T** | **H** | **P** | **T** | **H** | **P** | **T** | **H** | **P** | **T** | **H** | **P** | **T** | **H** | **P** | **T** | **H** | **P** | **T** | **H** | **P** | **T** | **H** | **P** | **T** |
| Razor-billed Curassow | n | 2 | 0 | 3 | 6 | 1 | 7 | 12 | 1 | 13 | 2 | 1 | 3 | 2 | 0 | 4 | 6 | 0 | 7 | 0 | 0 | 1 | 4 | 0 | 5 | 1 | 0 | 2 | **35** | **3** | **45** |
| *Mitu tuberosum* | *%* | 9 | 0 | 14 | 22 | 4 | 26 | 35 | 3 | 38 | 25 | 13 | 38 | 8 | 0 | 15 | 23 | 0 | 27 | 0 | 0 | 25 | 24 | 0 | 29 | 25 | 0 | 50 | **21** | **2** | **27** |
| Spix's Guan | n | 7 | 0 | 11 | 8 | 0 | 8 | 7 | 0 | 8 | 3 | 0 | 3 | 13 | 0 | 15 | 16 | 0 | 16 | 1 | 0 | 1 | 6 | 0 | 8 | 2 | 0 | 2 | **63** | **0** | **72** |
| *Penelope jacquacu* | *%* | 32 | 0 | 50 | 30 | 0 | 30 | 21 | 0 | 24 | 38 | 0 | 38 | 50 | 0 | 58 | 62 | 0 | 62 | 25 | 0 | 25 | 35 | 0 | 47 | 50 | 0 | 50 | **38** | **0** | **43** |
| Blue-throated Piping-guan | n | 0 | 0 | 0 | 5 | 0 | 7 | 2 | 1 | 4 | 1 | 0 | 1 | 0 | 0 | 0 | 1 | 0 | 1 | 2 | 0 | 2 | 0 | 0 | 0 | 0 | 0 | 0 | **11** | **1** | **15** |
| *Pipile cumanensis* | *%* | 0 | 0 | 0 | 19 | 0 | 26 | 6 | 3 | 12 | 13 | 0 | 13 | 0 | 0 | 0 | 4 | 0 | 4 | 50 | 0 | 50 | 0 | 0 | 0 | 0 | 0 | 0 | **7** | **1** | **9** |
| White-winged Trumpeter | n | 0 | 0 | 0 | 0 | 0 | 3 | 0 | 0 | 2 | 0 | 0 | 0 | 1 | 1 | 7 | 0 | 0 | 1 | 0 | 0 | 0 | 0 | 1 | 3 | 0 | 0 | 0 | **1** | **2** | **16** |
| *Psophia leucoptera* | *%* | 0 | 0 | 0 | 0 | 0 | 11 | 0 | 0 | 6 | 0 | 0 | 0 | 4 | 4 | 27 | 0 | 0 | 4 | 0 | 0 | 0 | 0 | 6 | 18 | 0 | 0 | 0 | **1** | **1** | **10** |
| Tinamus major | n | 0 | 0 | 8 | 1 | 0 | 2 | 1 | 2 | 7 | 0 | 0 | 1 | 0 | 0 | 0 | 0 | 1 | 1 | 0 | 0 | 0 | 0 | 0 | 1 | 0 | 0 | 0 | **2** | **3** | **20** |
| *Tinamous major* | *%* | 0 | 0 | 36 | 4 | 0 | 7 | 3 | 6 | 21 | 0 | 0 | 13 | 0 | 0 | 0 | 0 | 4 | 4 | 0 | 0 | 0 | 0 | 0 | 6 | 0 | 0 | 0 | **1** | **2** | **12** |
| **Total** | **n** | **9** | **0** | **22** | **20** | **1** | **27** | **22** | **4** | **34** | **6** | **1** | **8** | **16** | **1** | **26** | **23** | **1** | **26** | **3** | **0** | **4** | **10** | **1** | **17** | **3** | **0** | **4** | **112** | **9** | **168** |
|  | % | **41** | **0** | **100** | **74** | **4** | **100** | **65** | **12** | **100** | **75** | **13** | **100** | **62** | **4** | **100** | **88** | **4** | **100** | **75** | **0** | **100** | **59** | **6** | **100** | **75** | **0** | **100** | **67** | **5** | **100** |

NR: the year was not registered; H: *Haemoproteus* spp.; P: *Plasmodium* spp.; T: total of birds captured for that year or subtotal. Percentages were calculated and rounded for the total of that year or subtotal section.

**Table S2**. Closest related lineages to the new lineages recorded in this study identified by MalAvi Blast.

| **New lineages** | **Accession Number** | **MalAvi lineage** | **Sequence identity** | **Nucleotide correspondence** |
| --- | --- | --- | --- | --- |
| MITTUB02 | MZ614937 | NISCU2 | 97% | 457/469 |
| MITTUB03 | MZ614925 | TOFLA03 | 99% | 463/469 |
| MITTUB04 | MZ614930 | TOFLA03 | 99% | 462/469 |
| MITTUB05 | MZ614931 | ANIIGN01 | 99% | 463/469 |
| MITTUB06 | MZ614934 | TOFLA03 | 98% | 461/469 |
| MITTUB07 | MZ614935 | TOFLA03 | 99% | 464/469 |
| MITTUB08 | MZ614936 | TOFLA03 | 98% | 461/469 |
| MITTUB09 | MZ614927 | PENOBS01 | 98% | 460/469 |
| MITTUB10 | MZ614924 | TOFLA03 | 98% | 455/469 |
| MITTUB11 | MZ614926 | TOFLA03 | 97% | 457/469 |
| MITTUB12 | MZ614922 | PENOBS01 | 99% | 465/469 |
| MITTUB13 | MZ614932 | TOFLA03 | 99% | 463/469 |
| MITTUB14 | MZ614919 | TOFLA03 | 98% | 460/469 |
| MITTUB15 | ON246344 | ZOCAP09 | 95% | 444/469 |
| PENJAC02 | MZ614928 | ANIIGN01 | 99% | 462/469 |
| PENJAC03 | MZ614928 | PENOBS01 | 99% | 464/469 |
| PENJAC04 | MZ614928 | PENOBS01 | 99% | 462/469 |
| PENJAC05 | MZ614928 | ANIIGN01 | 99% | 467/469 |
| PIPCUM02 | MZ614928 | TOFLA03 | 99% | 467/469 |
| PIPCUM03 | MZ614928 | PENOBS01 | 99% | 463/469 |
| PIPCUM04 | MZ614928 | TOFLA03 | 99% | 466/469 |
| PSOLEU02 | MZ614928 | THACAE01 | 98% | 458/469 |
| PSOLEU03 | MZ614928 | POECAR03 | 96% | 448/469 |
| TINMAJ01 | MZ614928 | CRYUND01 | 96% | 452/469 |

**Table S3.** Biological variables included in regression models and criteria used for model selection to analyse parasite prevalence.

| **Model** | **Explanatory Variables** | **AIC** | **Δ AIC** | **AIC Weight** |
| --- | --- | --- | --- | --- |
| HB4 | Species | 148.6 | 0 | 0.54 |
| HB3 | Family | 149.81 | 1.21 | 0.3 |
| HB2 | Species + Sex | 151.85 | 3.26 | 0.11 |
| HB1 | Family + Sex | 153.26 | 4.66 | 0.05 |
| HB5 | Sex | 217.69 | 69.1 | 0 |
| PB2 | Species + Sex | 68.18 | 0 | 0.39 |
| PB4 | Species | 68.36 | 0.18 | 0.36 |
| PB1 | Family + Sex | 70.77 | 2.59 | 0.11 |
| PB3 | Family | 70.81 | 2.64 | 0.1 |
| PB5 | Sex | 72.76 | 4.58 | 0.04 |
| HB: models for *Haemoproteus* spp. infection; PB: models for *Plasmodium* spp. infection. AIC: Akaike’s Information Criterion. Models are ordered by lowest AIC value per group. | | | | |

**Table S4.** Ecological variables included in regression models and criteria used for model selection to analyse parasite prevalence.

| **Model** | **Explanatory Variables** | **AIC** | **Δ AIC** | **AIC Weight** |
| --- | --- | --- | --- | --- |
| HE21 | Fruits Forest | 184.99 | 0 | 0.42 |
| HE10 | Temperature + Fruits Forest | 186.91 | 1.92 | 0.16 |
| HE9 | Year + Fruits Forest | 186.92 | 1.93 | 0.16 |
| HE8 | Year + Temperature + Fruits Forest | 188.65 | 3.66 | 0.07 |
| HE23 | Season | 188.85 | 3.86 | 0.06 |
| HE16 | Temperature + Season | 190.73 | 5.74 | 0.02 |
| HE15 | Year + Season | 190.79 | 5.8 | 0.02 |
| HE22 | Fruits Swamp | 190.95 | 5.96 | 0.02 |
| HE20 | River Level | 191.59 | 6.6 | 0.02 |
| HE13 | Temperature + Fruits Swamp | 192.6 | 7.61 | 0.01 |
| HE14 | Year + Temperature + Season | 192.78 | 7.79 | 0.01 |
| HE7 | Temperature + River Level | 193.2 | 8.21 | 0.01 |
| HE6 | Year + River Level | 194.09 | 9.1 | 0 |
| HE19 | Precipitation | 194.92 | 9.93 | 0 |
| HE18 | Temperature | 194.99 | 10 | 0 |
| HE5 | Year + Temperature + River Level | 195.65 | 10.66 | 0 |
| HE12 | Year + Fruits Swamp | 196.13 | 11.14 | 0 |
| HE11 | Year + Temperature + Fruits Swamp | 196.59 | 11.6 | 0 |
| HE4 | Temperature + Precipitation | 196.82 | 11.83 | 0 |
| HE17 | Year | 198.83 | 13.84 | 0 |
| HE1 | Year + Temperature | 200.42 | 15.43 | 0 |
| HE3 | Year + Precipitation | 200.62 | 15.63 | 0 |
| HE2 | Year + Temperature + Precipitation | 202.36 | 17.37 | 0 |
| **Table S4.** Continued | | | | |
| PE19 | Precipitation | 67.24 | 0 | 0.49 |
| PE4 | Temperature + Precipitation | 69.17 | 1.93 | 0.19 |
| PE22 | Fruits Swamp | 70.95 | 3.71 | 0.08 |
| PE20 | River Level | 71.97 | 4.73 | 0.05 |
| PE21 | Fruits Forest | 72.2 | 4.96 | 0.04 |
| PE18 | Temperature | 72.3 | 5.06 | 0.04 |
| PE13 | Temperature + Fruits Swamp | 72.94 | 5.7 | 0.03 |
| PE3 | Year + Precipitation | 73.22 | 5.98 | 0.02 |
| PE23 | Season | 73.56 | 6.32 | 0.02 |
| PE7 | Temperature + River Level | 73.89 | 6.65 | 0.02 |
| PE10 | Temperature + Fruits Forest | 74.16 | 6.92 | 0.02 |
| PE2 | Year + Temperature + Precipitation | 75.21 | 7.97 | 0.01 |
| PE16 | Temperature + Season | 75.56 | 8.32 | 0.01 |
| PE17 | Year | 79.24 | 12 | 0 |
| PE1 | Year + Temperature | 80.24 | 13 | 0 |
| PE12 | Year + Fruits Swamp | 80.26 | 13.02 | 0 |
| PE15 | Year + Season | 81.08 | 13.84 | 0 |
| PE6 | Year + River Level | 81.14 | 13.9 | 0 |
| PE9 | Year + Fruits Forest | 81.17 | 13.93 | 0 |
| PE11 | Year + Temperature + Fruits Swamp | 81.41 | 14.17 | 0 |
| PE8 | Year + Temperature + Fruits Forest | 81.99 | 14.75 | 0 |
| PE5 | Year + Temperature + River Level | 82.23 | 14.99 | 0 |
| PE14 | Year + Temperature + Season | 82.99 | 15.75 | 0 |
| HE: models for *Haemoproteus* spp. infection; PE: models for *Plasmodium* spp. infection; AIC: Akaike’s Information Criterion. Precipitation and river level values were obtained from the four closest stations; seasons were classified as rainy for months with a precipitation average over +1SD (January and March), intermediate within ± 1 SD (February, May-June, October-December) and dry under -1 SD (July-September); temperature was averaged monthly; the monthly percentage of ripe fruit in two habitats (upland forest and swamp forest) was taken from Bizri et al. (2018). Models are ordered by lowest AIC value per group. | | | | |

**Table S5.** Ecological variables included in regression models and criteria used for model selection to analyse the number of *Haemoproteus* spp. lineages recorded.

| **Model** | **Explanatory Variables** | **AICc** | **Δ AICc** | **AICc Weight** |
| --- | --- | --- | --- | --- |
| LE19 | Precipitation | 154.69 | 0 | 0.35 |
| LE20 | River Level | 156.88 | 2.19 | 0.12 |
| LE22 | Fruits Swamp | 156.88 | 2.19 | 0.12 |
| LE4 | Temperature + Precipitation | 157.2 | 2.51 | 0.1 |
| LE21 | Fruits Forest | 157.3 | 2.61 | 0.09 |
| LE18 | Temperature | 157.49 | 2.8 | 0.09 |
| LE23 | Season | 159.29 | 4.6 | 0.03 |
| LE7 | Temperature + River Level | 159.33 | 4.64 | 0.03 |
| LE13 | Temperature + Fruits Swamp | 159.41 | 4.72 | 0.03 |
| LE10 | Temperature + Fruits Forest | 159.81 | 5.12 | 0.03 |
| LE16 | Temperature + Season | 161.96 | 7.27 | 0.01 |
| LE17 | Year | 171.78 | 17.09 | 0 |
| LE3 | Year + Precipitation | 172.05 | 17.36 | 0 |
| LE2 | Year + Temperature + Precipitation | 174.39 | 19.7 | 0 |
| LE6 | Year + River Level | 174.92 | 20.23 | 0 |
| LE1 | Year + Temperature | 175.15 | 20.46 | 0 |
| LE12 | Year + Fruits Swamp | 175.18 | 20.49 | 0 |
| LE9 | Year + Fruits Forest | 175.27 | 20.58 | 0 |
| LE5 | Year + Temperature + River Level | 178.6 | 23.91 | 0 |
| LE11 | Year + Temperature + Fruits Swamp | 178.66 | 23.97 | 0 |
| LE8 | Year + Temperature + Fruits Forest | 178.95 | 24.26 | 0 |
| LE15 | Year + Season | 179.01 | 24.32 | 0 |
| LE14 | Year + Temperature + Season | 182.15 | 27.46 | 0 |
| LE: models for *Haemoproteus* spp. number of lineages; AICc: Akaike’s Information Criterion of second order. Precipitation and river level values were obtained from the four closest stations; seasons were classified as rainy for months with a precipitation average over +1SD (January and March), intermediate within ± 1 SD (February, May-June, October-December) and dry under -1 SD (July-September); temperature was averaged monthly; the monthly percentage of ripe fruit in two habitats (upland forest and swamp forest) was taken from Bizri et al. (2018). Models are ordered by lowest AICc value. | | | | |

**Table S6.** Variables included in regression models and criteria used for model selection to analyse the influence of sampling method and sample preservation on parasite detection.

| **Model** | **Explanatory Variables** | **AIC** | **Δ AIC** | **AIC Weight** |
| --- | --- | --- | --- | --- |
| C5 | Card Type + Sample Preservation | 207.22 | 0 | 0.34 |
| C4 | Card Type + Sample Preservation + Fungal Presence | 207.82 | 0.6 | 0.25 |
| C1 | Card Type | 207.86 | 0.64 | 0.25 |
| C6 | Card Type + Fungal Presence | 208.91 | 1.69 | 0.15 |
| C2 | Sample Preservation | 215.75 | 8.53 | 0 |
| C7 | Sample Preservation + Fungal Presence | 215.92 | 8.7 | 0 |
| C3 | Fungal Presence | 218.01 | 10.79 | 0 |
| C: models for the sample method and preservation condition. AIC: Akaike’s Information Criterion. Card type: Filter paper W3, FTA® cards and Protein saver cards; sample preservation: good, medium, bad; fungal presence: abundant, present, scarce, none. Models are ordered by lowest AIC value. | | | | |
